# Relation of perceptions of educational environment with mindfulness among Chinese medical students: a longitudinal study

**DOI:** 10.3402/meo.v21.30664

**Published:** 2016-04-25

**Authors:** Xin Xu, Daxing Wu, Xiaohua Zhao, Junxiang Chen, Jie Xia, Mulei Li, Xueqing Nie, Xue Zhong

**Affiliations:** 1 Medical Psychological Institute, The Second Xiangya Hospital of Central South University, Changsha P.R. China; 2 Medical Education Office, The Second Xiangya Hospital of Central South University, Changsha, P.R. China

**Keywords:** perceptions, educational environment, mindfulness, medical students, hierarchical multiple regression, predicted effect

## Abstract

**Background:**

Perceived educational environment influences academic outcomes, such as academic achievement, students’ behaviors, well-being, socio-emotional adjustment and explicit self-esteem. Mindfulness is a set of skills that are beneficial to physical and mental health. Recently, it has been increasingly discussed about its usefulness in education, but little research has explored whether mindfulness can predict perceptions of educational environment. The aim of this study was to explore Chinese medical students’ perceptions of learning environment and their relationship with mindfulness.

**Methods:**

Medical students at the Second Xiangya Hospital of Central South University (*N*=431) completed the Chinese version of Dundee Ready Educational Environment Measure (DREEM-C) and the Kentucky Inventory of Mindfulness Skills (KIMS-C). One year later, a subgroup of the cohort (*N*=231) completed the DREEM-C again. Independent-samples *t*-test, variance analysis, correlation analysis, and hierarchical multiple regression (HMR) were conducted.

**Results:**

DREEM-C total and subscales scores were net positive, but with room for improvement. Perceptions differed in relation to gender, academic year, and age. KIMS-C scores correlated with DREEM-C scores. The predictive effect persisted 1 year later.

**Conclusions:**

Medical students had net-positive perceptions about their learning environment. Higher mindfulness scores were associated with greater satisfaction with the environment and this association showed persistence.

The levels of expertise achieved by medical students play a vital role in other people's health and quality of life. The World Federation for Medical Education has emphasized the importance of the learning environment on student outcomes (1). Hence, for the good of broader society as well as medical students themselves, it is important that medical students are immersed in a satisfactory learning environment.

Educational environment can be defined as everything in the milieu in which an educational institution exists, including atmosphere, culture, values, resources, and social networks, as well as organizational, instructional, and interpersonal dimensions (2). Numerous studies have shown that school climate, as perceived by students, is a key factor in students’ outcomes, such as academic achievement, behaviors, well-being, socio-emotional adjustment, and explicit self-esteem (3–7). However, perceptions of a school's climate may differ across individuals.

The concept of mindfulness originates from Buddhist meditation practice (8). It can be defined briefly as one's moment-to-moment awareness of what is taking place now and accepting the present situation non-judgmentally (9, 10). Mindfulness requires people to devote their attention to their present setting and activity, rather than being caught up in fretting about the past or worrying about the future (11). It has been credited with cultivating clear thinking and openness to experience (12). Furthermore, mindfulness has been reported to have health benefits, both physically and mentally, including alleviation of chronic pain (13), stress (14), and depression (15), mitigation of burnout experiences (16), and recovery from eating disorders (17). Interest has developed in mindfulness as a clinical tool for the treatment of emotional distress as well as of bodily disease. More recently, potential application in education has been explored (18). For example, mindfulness has been reported to enhance students’ knowledge retention (19) and to improve students’ grades in reading and science (20), as well as their performance on high-stakes exams (21). Thus far, little attention has been paid to the question of how mindfulness may improve students’ perceptions of educational climate.

The aim of this study was to explore the characteristics of Chinese medical students’ perceptions of their learning environments and to examine whether their mindfulness aptitudes are associated with greater satisfaction with their environments. Finally, we also tested whether mindfulness index scores continue to predict perceptions 1 year later.

## Methods

### Participants

Using a cluster sampling method, 463 students majoring in medicine at the Second Xiangya Hospital of Central South University in Changsha were enrolled in this study. Subjects were excluded from the data analysis if >1/3 of the questionnaire items were unanswered or if the answers showed improbable regularity. Of the 463 questionnaires distributed, 431 were returned properly (not leading to exclusion), including 147, 191, and 93 from year 3, year 4, and year 5 students, respectively. The sample consisted of 171 men and 260 women, ranging in age from 18 to 24 years, with an average age of 21.47 years [standard deviation (SD)=0.94 years]. After 1 year, 231 participants from this cohort were recruited to complete the Dundee Ready Educational Environment Measure (DREEM) to assess long-term persistence of meditation training effects.

### Instruments

#### Chinese version of Dundee Ready Educational Environment Measure

The DREEM is a widely used instrument designed to measure clinical learning environment and educational atmosphere (22). The Chinese version of Dundee Ready Educational Environment Measure (DREEM-C) was translated from English into Chinese by two psychology postgraduates, back-translated into English by an English postgraduate to confirm accuracy of translation, and finally revised by a clinical psychologist. The Cronbach's α of the DREEM-C in this study was 0.92.

The DREEM-C consists of 50 items distributed among five domains: students’ perceptions of learning (SPL), students’ perceptions of teachers (SPT), students’ academic self-perceptions (SASP), students’ perceptions of atmosphere (SPA), and students’ social self-perceptions (SSSP). Of the 50 items, 41 describe educational climate directly and are scored from 0 (strongly disagree) to 4 (strongly agree). The remaining nine items are opposite to direct statements and are reversed scored. The maximum score is 200 points with higher scores representing greater satisfaction with one's educational environment.

#### Chinese version of Kentucky Inventory of Mindfulness Skills

The Chinese version of Kentucky Inventory of Mindfulness Skills (KIMS-C) was developed from the original KIMS with a back-translation procedure (23). The Cronbach's α for the KIMS-C in this study was 0.72. The KIMS-C is a 39-item self-report scale that assesses four mindfulness skills: observing (12 items), describing (8 items), acting with awareness (10 items), and accepting without judgment (9 items). Response options range from 1 (never or very rarely true) to 5 (almost always or always true). Items describing an absence of mindfulness are reversed scored. Higher total scores reflect greater mindfulness.

### Procedure

The questionnaires were handed out by psychology major students with prior research experience. Participants were informed about this study and took part voluntarily without any compensation. The trained experimenters introduced the inventories to the volunteers carefully according to the uniform instructions. The questionnaires were completed and returned to the experimenters immediately. One year later, a subset of 231 participants completed the DREEM-C again.

### Statistical analysis

Independent-samples t test and variance analysis were used to compare scores between demographic subgroups. The correlation analysis was used to determine whether DREEM-C scores correlated with mindfulness measures. Hierarchical multiple regression (HMR) analyses were performed to examine variable relationships while controlling the effects of covariates (gender, age, and program year) on correlation results. To assess the exact degree that mindfulness predicted student perception, covariates were entered in step 1 and independent variables (four mindfulness dimensions) were entered in step 2 stepwise. All statistical analyses were carried out with SPSS17.0.

## Results

### Relation of demographic characteristics to perceptions of educational environment

Mean (±SD) DREEM-C total and subscales scores, stratified by gender, year in program, and age subgroup, are reported in Table 1. We found that students’ perceptions, as reflected by their DREEM-C scores, were significantly affected by gender, program year, and age. Briefly, women scored higher than men. Year 4 students scored higher than year 3 and year 5 students. Students who were <21 years old were more satisfied than older students. According to the DREEM guide (24), the mean overall DREEM-C score (Table 1) was consistent with students feeling more positive than negative about their learning environment in general. Additionally, SPL and atmosphere subscores were in positive ranges (scores of 25–36), as well as SASP (scores of 17–24). Students perceived teachers as moving in the right direction (SPT scores of 23–33). Their social self-perception was reported to be only fair (SSSP scores of 15–21).

**Table 1:** Educational environment perception as indexed by mean DREEM-C scores (±SD) stratified by demographic characteristics

	DREEM-C subscale scores					
		
Group	SPL	SASP	SPT	SPA	SSSP	DREEM-C total score
All subjects	31.68±5.51	20.45±3.94	32.72±4.97	32.04±5.35	17.93±3.62	134.82±19.90
Gender						
Male	31.10±5.62	20.28±4.09	31.95±5.13	31.34±5.55	17.43±3.80	132.11±20.50
Female	32.06±5.41	20.57±3.84	33.22±4.81	32.50±5.18	18.26±3.47	136.61±19.34
*t*	−1.77	−0.74	−2.60**	−2.22*	−2.32*	−2.30*
Age						
≤21 years	32.20±5.62	20.82±3.99	33.32±4.86	32.44±5.55	17.91±3.87	136.69±20.65
>21 years	31.09±5.33	20.03±3.85	32.03±5.02	31.59±5.10	17.96±3.33	132.71±18.84
t	2.10*	2.08*	2.69**	1.65	−0.15	2.08*
Year in program						
3	30.89±5.56	19.86±4.30	31.49±5.13	30.70±5.52	17.47±3.56	130.40±20.83
4	32.56±5.24	20.78±3.70	34.25±4.36	32.86±5.24	18.12±3.70	138.56±18.91
5	31.14±5.76	20.72±3.76	31.49±5.09	32.49±4.93	18.28±3.52	134.13±19.07
F	4.45*	2.56	17.62**	7.40**	1.90	7.26**

DREEM-C: Chinese version of Dundee Ready Educational Environment Measure; SPL: students’ perceptions of learning; SASP: students’ academic self-perceptions; SPT: students’ perceptions of teachers; SPA: students’ perceptions of atmosphere; SSSP: students’ social self-perceptions.

* *p*<0.05,

** *p*<0.01.

### Correlation analysis

As shown in Table 2, DREEM-C total scores correlated positively with KIMS-C total scores (*r*=0.36, *p*<0.01). Of the KIMS-C subscales, only the describing and the acting with awareness subscales correlated with all aspects of students’ perceptions of their educational milieu, as indexed by DREEM-C scores.

**Table 2:** Correlations between DREEM-C and mindfulness assessment scores

	DREEM-C scores					
	
Mindfulness assessment	Total	SPL	SASP	SPT	SPA	SSSP
KIMS-C	0.36**	0.29**	0.32**	0.29**	0.33**	0.31**
Observe	0.09	0.11*	0.12*	0.06	0.06	0.06
Describe	0.38**	0.31**	0.30**	0.32**	0.35**	0.30**
AWA	0.37**	0.27**	0.30*	0.31**	0.37**	0.32**
AWJ	−0.09	−0.11*	−0.08	−0.09	−0.08	−0.03

DREEM-C: Chinese version of Dundee Ready Educational Environment Measure; SPL: students’ perceptions of learning; SASP: students’ academic self-perceptions; SPT: students’ perceptions of teachers; SPA: students’ perceptions of atmosphere; SSSP: students’ social self-perceptions; KIMS-C: Chinese version of Kentucky Inventory of Mindfulness Skills; AWA: act with awareness; AWJ: accept without judgment.

* *p<*0.05,

** *p*<0.01.

### Hierarchical multiple regression

The *R*, adjusted *R* ^2^, change in *R* ^2^ (Δ*R* ^2^), and standardized regression coefficient (β) values obtained in the HMR analysis of the influence of mindfulness skills on DREEM-C total scores are reported in Table 3. The statistical HMR analysis values related to the predictive capacity of mindfulness for each dimension of perceived educational environment in the DREEM-C are shown in Table 4. Briefly, with the exception of observing, the remaining three KIMS-C factors were significant predictors of learning environment perception. Describing, acting with awareness, and accepting without judgment explained 21% of the variability in DREEM-C scores, with a particularly strong relationship to the SPA subscale (Δ*R* ^2^=0.19).

**Table 3:** HMR of mindfulness in relation to perceptions of educational environment

	DREEM-C			
	
Predictor	*R*	Adjusted *R* ^2^	Δ*R* ^2^	*β*
Step 1	0.21	0.04	0.05**	
Gender				0.07
Age				−0.19**
Grade				0.18**
Step2	0.51	0.25	0.21**	
Observe				−
Describe				0.25**
AWA				0.32**
AWJ				−0.17**

HMR: hierarchical multiple regression; DREEM-C: Chinese version of Dundee Ready Educational Environment Measure; AWA: act with awareness; AWJ: accept without judgment.

* *p*<0.05,

** *p*<0.01.

**Table 4:** HMR of mindfulness to each dimension of educational environment perception in the DREEM-C

	SPL		SASP		SPT		SPA		SSSP	
					
Predictor	Δ*R* ^2^	*β*	Δ*R* ^2^	*β*	Δ*R* ^2^	*β*	Δ*R* ^2^	*β*	Δ*R* ^2^	*β*
Step 1	0.02*		0.03**		0.04**		0.05**		0.03**	
Gender		0.05		0.00		0.08		0.06		0.09
Age		−0.14*		−0.17**		−0.19**		−0.18**		−0.10
Grade		0.10		0.17**		0.13*		0.22**		0.13*
Step2	0.14**		0.15**		0.15**		0.19**		0.14**	
Observe		−		−						
Describe		0.22**		0.20**		0.21**		0.22**		0.20**
AWA		0.23**		0.27**		0.27**		0.32**		0.27**
AWJ		−0.16**		−0.14**		−0.15**		−0.16**		−0.10*

HMR: hierarchical multiple regression; DREEM-C: Chinese version of Dundee Ready Educational Environment Measure; SPL: students’ perceptions of learning; SASP: students’ academic self-perceptions; SPT: students’ perceptions of teachers; SPA: students’ perceptions of atmosphere; SSSP: students’ social self-perceptions; AWA: act with awareness; AWJ: accept without judgment.

* *p*<0.05,

** *p*<0.01.

The most powerful predictor of mindfulness was acting with awareness (*β*=0.32, 0.23, 0.27, 0.27, 0.32, and 0.27 for DREEM-C total, SPL, SASP, SPT, SPA, and SSSP, respectively, *p*<0.01). Contrary to our expectations, we found that accepting without judgment was a significant inverse predictor of perceived educational environment (*β*= − 0.17, −0.16, −0.14, −0.15, −0.16, and −0.10, respectively). Furthermore, with covariates, independent variables, and methods held fixed, describing alone accounted for 6% of the variability in perceptions of educational environment in the 1-year follow-up DREEM-C scores (*N*=231; *R*=0.29, Δ*R* ^2^=0.06, *β*=0.26, *p*<0.01).

## Discussion

The present study of the characteristics of medical students’ perceptions of educational environment and their relation to mindfulness showed that the students’ DREEM-C total and subscales scores were all above average, but with room for improvement. The findings of this study complement previous work revealing that mindfulness can improve school climate for students (25).

Our finding that fourth-year medical students’ scores indicated greater satisfaction with the environment than those of students at other levels may be due to fourth-year students being in a clinical novitiate period. Before the fourth year, students are taught primarily in classroom and have little contact with patients. During the novitiate period, they are taught by clinicians rather than academics; they are given practical experience in a hospital setting, rather than wrote knowledge from books, which they tend to view as very helpful for preparing them for their future work. By year 5, medical students have likely habituated to the clinical environment.

Strong mindfulness index scores were predictive of positive perceptions of educational environment, even a year later, suggesting that mindfulness is a stable factor in predicting environmental perception. However, the predictive power was limited. This relationship could be due to the more mindful students with better developed acting with awareness skills being more absorbed in learning activities than others. In addition, mindfulness refers to the ability to experience the present situation consciously. Generally, students must be attentive to the information given by their teachers to perform very well academically. These results confirm prior validation studies’ suggestion that mindfulness is associated with better academic performance (19–21). Generally speaking, mindful students are likely to be well received by their peers and teachers. Thus, experiencing a pleasant social atmosphere, such students would be expected to perceive their educational environment as harmonious and satisfying. Furthermore, mindfulness involves a state of being open and accepting of experiences. Thus even if there are weaknesses, compared with captious students, mindful students would be expected to adopt a relatively open and tolerant attitude to their surroundings, contributing to a more positive perceptions of the environment.

Our finding that accepting without judgment was a negative predictor of satisfaction with one's educational environment was unexpected. It could be that lack of evaluation could result in students not differentiating much between positive and negative experiences. Hence, in this context, a lack of active judgment might hamper students’ perceptions of their learning environment.

There are some limitations of this study. First, we only assessed medical students in one school, which limits the generalizability of the results to some degree. The present study questions should be examined further in more broadly representative samples. Second, we only reported relationships between students’ perceptions of educational environment and mindfulness, without developing specific advice as to how to increase students’ mindfulness. Future work should explore mindfulness training methods and to what extend they are suitable and effective for medical students.

## Conclusions

Medical students at the Second Xiangya Hospital of Central South University in Changsha had a net-positive perception of their learning environment. Mindfulness can be a small, but significant predictor of students’ perceptions of their learning environment and the predictive capacity can be sustained for a full year. Given that mindfulness is an inherent ability that all people can develop (12), medical students’ may benefit from instruction in mindfulness skills, especially in the areas of describing and acting with awareness, alongside the development of their professional skills.
